# Effects of Balance-Based Exergame Training With Variable Difficulty on Balance and Spatiotemporal Gait Outcomes in Adults With Mild Cognitive Impairment: Randomized Controlled Trial

**DOI:** 10.2196/74092

**Published:** 2025-11-18

**Authors:** Aruba Saeed, Imran Amjad, Imran Khan Niazi, Abdullah Alzahrani, Muhammad Shafiq, Heidi Haavik

**Affiliations:** 1 Riphah International University Rawalpindi, Pakistan; 2 Lahore University of Biological and Applied Sciences Lahore Pakistan; 3 Centre for Chiropractic Research, New Zealand College of Chiropractic Auckland, New Zealand; 4 Faculty of Health & Environmental Sciences, Health & Rehabilitation Research Institute, AUT University Auckland New Zealand; 5 Department of Health Rehabilitation Sciences, College of Applied Medical Sciences, Shaqra University Riyadh Saudi Arabia

**Keywords:** balance, cognitive load, difficulty, exergaming, gait, mild cognitive impairment, walking

## Abstract

**Background:**

Exergame balance training integrates cognitive and motor challenges, potentially enhancing neuroplasticity, postural control, and gait stability in mild cognitive impairment (MCI). However, whether modulating the task difficulty of a balance-based exergame training may influence posture- and gait-related outcomes remains unknown.

**Objective:**

We compared balance and gait improvements across exergame training groups performing exercise with different difficulty levels and a Wii Fit group in adults with MCI.

**Methods:**

This 4-armed, parallel design, double-blinded, randomized clinical trial included 97 participants with MCI (Montreal Cognitive Assessment score=18-25). Participants were convenience-sampled from the Railway General Hospital, Rawalpindi, Pakistan, and randomized to one of 4 intervention groups: low-difficulty, moderate-difficulty, high-difficulty exergame training or a Wii Fit training group. Each participant completed 24 sessions (40 min, 3/week) supervised by physical therapists. Gait and balance were assessed using time up and go (TUG), cognitive time up and go (C-TUG), and the Gait & Balance mobile app at baseline and after 4 and 8 weeks. Although the calculated sample size was 80, 97 were recruited to offset attrition. Eighty-seven participants completed the study (94% adherence) (attrition: low-difficulty 1, moderate 3, high 2, Wii Fit 2; 10% total). Data were analyzed using mixed-model analysis of covariance with baseline values as covariates to assess time×group interactions. Bonferroni-adjusted post hoc comparisons revealed between-group differences.

**Results:**

High-difficulty training showed the greatest TUG gains (–0.71, SD 0.32; *P*=.03, anteroposterior (AP) steadiness with eyes open (EO) on firm surface (0.04, SD 0.02; *P*=.04), step time variability head forward (HF; 0.06, SD 0.09; *P*=.02), walking speed HF (0.08, SD 0.04; *P*=.05), step time head turn (HT; –0.04, SD 0.02; *P*=.04), step time variability HT (–0.35, SD 0.09; *P*<.001), step length variability (–0.27, SD 0.13; *P*=.04), and walking speed HT (0.09, SD 0.04; *P*=.01) versus Wii Fit. Moderate-difficulty training improved AP steadiness EO firm (0.05, SD 0.02; *P*=.03) and reduced step time variability HT (–0.26, SD 0.09; *P*=.01). Low-difficulty training improved C-TUG (–1.61, SD 0.63; *P*=.01), AP steadiness EO firm (0.05, SD 0.02; *P*=.03), step time variability HF (–0.20, SD 0.09; *P*=.03), step time variability HT (–0.25, SD 0.09; *P*=.01), step length variability (–0.31, SD 0.12; *P*=.014), and walking speed HT (0.11, SD 0.04; *P*=.03). No significant differences observed between exergame difficulty groups (*P*>.05).

**Conclusions:**

Balance-based exergame training improves balance and gait in adults with MCI, with no significant differences across difficulty levels, while the high- and low-difficulty training outperformed Wii Fit in several outcomes. High-difficulty training yielded the most consistent improvements in TUG, postural steadiness, gait variability, and walking speed. These results support graded cognitive-motor exergaming as an effective strategy for enhancing postural control and walking stability in MCI, potentially aiding fall prevention and mobility preservation in aging populations.

**Trial Registration:**

ClinicalTrials.gov NCT04959383; https://clinicaltrials.gov/study/NCT04959383

## Introduction

Aging is associated with a decline in both physical and cognitive abilities [1], increasing the risk of falls, which is one of the leading causes of morbidity, mortality, and functional dependence among older adults [2]. Neuromuscular degeneration, sensory impairments, and executive function deficits contribute to balance and gait dysfunction, further elevating fall risk in older adults [3]. Importantly, age-related neurodegenerative changes can accelerate cognitive decline, with aging being one of the strongest risk factors for developing mild cognitive impairment (MCI) and dementia [3]. MCI is a clinical condition that represents an intermediate stage between normal cognitive aging and dementia [4]. It is defined by cognitive decline greater than expected for age and education, which may affect complex tasks but does not significantly impair independence [4-6]. Individuals with MCI are at an increased risk of progressing to dementia, a condition marked by more severe cognitive decline that interferes with daily functioning [4-6]. Beyond cognitive changes, MCI is increasingly recognized for its motor implications, with impairments in gait and balance being among the earliest motor manifestations [3,7] contributing to a more than 2-fold increase in fall risk and up to 4 times the rate of recurrent falls compared with cognitively healthy peers [8]. The risk is further amplified in dementia, where individuals experience a 2- to 8-fold greater likelihood of falls relative to their cognitively intact peers [9]. Gait alterations, including slower walking speed, increased stride variability, and reduced balance, are well-established predictors of future falls [10]. Given that 17.3% of adults aged 60 years and older are affected by MCI (95% CI 13.8%-20.8%) [11], and the annual incidence is 1%-2% in the general population [12]. The growing number of affected individuals highlights the urgent need for targeted interventions that address gait and balance to reduce fall risk and maintain functional independence [7].

Cognitive functions are integral to balance and gait, brain regions associated with specific cognitive processes, such as memory and executive functioning, also play a key role in motor control (eg, balance and gait) [13]. MCI is associated with atrophy and hypometabolism in the prefrontal cortex and hippocampus, as well as disrupted fronto-striatal and parietal networks [14]. These regions are crucial for executive function, memory, visuospatial processing, and motor planning [14]. Such neural alterations impair cognitive-motor integration, leading to deficits in dual-task performance and manifesting as balance instability and gait disturbances [15,16]. Motor and cognitive areas are functionally interconnected through extensive neural networks that integrate executive processing with locomotor control [13]. The prefrontal cortex, which governs higher-order cognitive functions such as attention, planning, and decision-making, communicates bidirectionally with subcortical structures, including the basal ganglia and limbic system [17], as well as locomotor centers such as the subthalamic locomotor region, mesencephalic locomotor region, and cerebellar locomotor region [18]. This integration allows cognitive processes to influence motor planning, postural adjustments, and adaptive responses to environmental demands [17,18]. When cognitive domains such as judgment, reaction time, and task switching become impaired, the prefrontal cortex’s regulatory influence on these motor circuits is diminished [19,20]. Consequently, the efficiency of motor execution is disrupted, leading to deficits in gait initiation, reduced walking speed, impaired postural stability, and increased stride variability, which collectively heighten the risk of imbalance and falls [21]. Given the strong interplay between cognition and motor function, interventions targeting both domains simultaneously may offer substantial benefits, including enhanced cognitive processing, neuroplasticity, improved balance, gait speed, and functional mobility [22].

Exergaming is a combination of physical exercise with interactive gaming technology, in which users perform real-life body movements to control game actions through motion sensors, balance boards, or camera-based systems [23-25]. By blending structured physical activity with engaging digital environments, exergaming has gained prominence as a rehabilitation tool, offering an enjoyable and motivating way to promote regular exercise and therapeutic training [26]. Unlike traditional training, it delivers real-time multisensory feedback, demanding simultaneous visual attention, decision-making, and motor coordination [27]. This multimodal approach engages users in concurrent physical and cognitive tasks, which promote skilled learning, provide cognitive stimulation, and are associated with neuroplastic adaptations in key brain regions, including the frontal cortex, hippocampus, and parietal areas [24,28]. Importantly, exergaming has shown promise in improving postural stability, balance, and functional mobility in individuals with MCI [29]. The mechanism is likely multifactorial: exergames require continuous weight shifting and postural adjustments, which train the vestibular system and proprioceptive feedback loops, leading to improved balance control [30]. Simultaneously, responding to visual and auditory cues enhances sensorimotor integration and reaction speed [25,29]. The cognitive demands of exergaming, such as divided attention, decision-making, and task switching, activate executive function networks in the prefrontal cortex, which are strongly linked to gait regulation [25,29,30]. Repeated dual-task practice strengthens neural pathways between motor and cognitive centers (eg, frontal cortex, cerebellum, and basal ganglia), fostering neuroplastic changes that support better gait speed, stride regularity, and stability [30]. Collectively, these adaptations help reduce fall risk and improve walking confidence in this population [24,26,31]. However, the role of task difficulty and graded cognitive-motor challenge in optimizing these balance and gait outcomes remains underexplored.

Integrating balance exercises into exergaming may enhance motor and cognitive function by engaging key neural structures such as the vestibular system, cerebellum, hippocampus, and prefrontal cortex [32]. Beyond these neural effects, balance training improves postural control, proprioceptive feedback, and lower-limb strength, leading to better weight shifting, stride regularity, and stability during walking [33]. These adaptations contribute to measurable improvements in gait speed, step length, and dynamic balance, which are critical for reducing fall risk in individuals with MCI [33,34]. Notably, the effectiveness of balance training is strongly linked to task complexity, as higher attention demands typically foster greater cognitive-motor engagement [35]. The “Guided Plasticity Facilitation Framework” suggests that continuously challenging tasks promote ongoing neural adaptations, strengthening cognitive-motor integration and improving coordination [24,36]. These benefits are further amplified when balance training is conducted on an unstable surface, which increases cognitive-motor engagement and enhances vestibular system activation compared with stable surfaces [37].

Although exergaming has been studied for fall prevention and postural control in MCI [29], the effect of graded cognitive-motor challenge in optimizing training effects on balance and gait remains unexplored. Addressing this gap is essential to better understand how exergaming can be optimized to enhance gait and balance performance, ultimately contributing to fall prevention strategies in MCI.

This study introduces a novel exergame with an adjustable difficulty setting designed to systematically modulate cognitive load and motor demands. Previous findings support the efficacy of this exergame in delivering graded cognitive challenges [38]. The difficulty levels were modified by adjusting the goal area and ball speed. To further enhance cognitive-motor interactions, training was conducted on a wobble board, introducing postural instability that necessitated continuous balance adjustments, thereby amplifying cognitive-motor engagement.

This randomized clinical trial (NCT04959383) was designed with preplanned outcomes, with our previous paper focusing on executive function improvements in response to exergaming [39]. This study examines a separate objective: comparing the effects of balance-based exergame training with progressive difficulty levels on unstable surfaces against both other experimental groups and with the exergame balance training on stable surfaces (Wii Fit). Given the greater cognitive-motor demands of progressively challenging tasks, we hypothesize that exergame training with increasing difficulty on unstable surfaces will result in greater improvements in balance and spatiotemporal gait parameters.

## Methods

### Design and Participants

This 4-arm, parallel design randomized clinical trial was conducted at Railway Hospital in Rawalpindi, Pakistan, from September 2021 to December 2022, with participants randomly allocated in a 1:1:1:1 ratio to one of the 4 intervention groups. The methodology follows the Consolidated Standards of Reporting Trials (CONSORT) 2010 [40] guidelines for randomized trials and the CONSORT-EHEALTH (Consolidated Standards of Reporting Trials of Electronic and Mobile Health Applications and Online Telehealth) checklist for reporting exergame interventions [41], with the completed checklists provided as Multimedia Appendices 1 and 2.

We have structured the description of both the intervention and control conditions using the Consensus on Exercise Reporting Template. The interventions aligned with the Helsinki Declaration of Ethical Principles for Medical Research. Participants were recruited through 2 medical camps and community advertisements, including radio announcements (FM 102.2) and printed flyers. Follow-up telephone calls were conducted to ensure participant compliance with the intervention protocol.

The participants were male and female aged 60-75 years who met the Petersen/Winblad criteria for MCI [42] and had a Montreal Cognitive Assessment (MoCA) score of 18-25 (Urdu Version) [43], consistent with more recent literature defining the MCI range, which slightly extends below the range (20-24) specified in the trial registry. Participants underwent a medical screening to confirm exercise safety. Those with controlled metabolic or cardiovascular conditions (eg, well-managed hypertension or type 2 diabetes) cleared by their physician were eligible. Additional inclusion criteria required participants to have the ability to read and write in Urdu, adequate vision for engaging in physical and exercise training, and a balance score of 20-25 (mild balance impairment category) on the Mini-Balance Evaluation Systems Test (Mini-BESTest), ensuring that participants had sufficient baseline balance capacity to safely perform exergame tasks. As the intervention involved the exergame technology, the ability to understand and follow the exergame requirements was considered necessary and thus served as an implicit eligibility criterion. Participants involved in any other exercise of >150 min/week, impaired mobility, history of dizziness, vertigo, clinically significant psychiatric, other neurological, unstable or severe cardiovascular, metabolic, or orthopedic conditions were excluded [44]. At baseline, demographic information (age and sex) and clinical measures (BMI and Mini-BESTest scores) were collected for all participants before randomization. Age and sex were recorded through participant self-report and confirmed against medical records where available. Height and weight were measured using a standardized stadiometer and calibrated digital weighing scale, and BMI was calculated as weight in kilograms divided by height in meters squared (kg/m²). The Mini-BESTest was administered by trained physical therapists following standardized procedures to assess baseline balance function.

### Ethical Considerations

**Figure 1:**
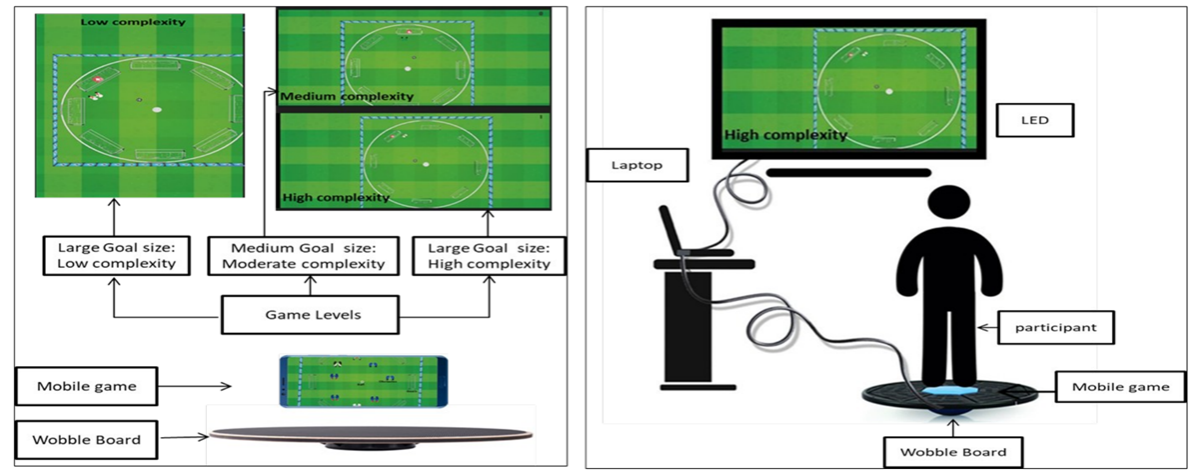
(A) Image of the exergaming interface used in the intervention group, illustrating the balance-based training tasks. (B) Experimental setup showing a participant performing exergame-based balance training. These images represent the intervention arm of a randomized controlled trial conducted in adults with mild cognitive impairment [39]. LED: light-emitting diode.

The study protocol was reviewed and approved by the Riphah University Ethical Review Committee (approval number Riphah/RCRS/REC/1954) and was registered at ClinicalTrials.gov (NCT04959383). All participants were provided with detailed information regarding the study’s objectives, procedures, potential risks, and benefits. They had the opportunity to ask questions, and written informed consent was obtained before participation, ensuring their voluntary involvement. To maintain privacy and confidentiality, all data were anonymized and deidentified before analysis. No identifiable images of participants are included in this manuscript. Participants did not receive any financial compensation for their participation. The overall process of participant enrolment, randomization, follow-up, and dropout was documented according to CONSORT guidelines, with the flow diagram presented in the Results section (Figure 1).

### Sample Size

The sample size was calculated using G-Power software (Kiel University, DE) [45], based on a Cohen *f* effect size of 0.38 for MoCA [46]. Using a 95% confidence level (α=0.05) and 80% power, with 4 groups and one covariate, the minimum required sample size was 80 participants. Accounting for an expected attrition rate of 20%, a total of 97 patients were recruited.

### Randomization and Blinding

Participants were selected through a nonprobability convenience sampling technique and randomized using a lottery method. To ensure allocation concealment, 100 sealed opaque envelopes (25 per group) containing prelabeled group assignments were prepared in advance by the principal investigator. Participants selected an envelope only after completing all preintervention assessments, preventing knowledge of allocation prior to baseline testing. The principal investigator generated the allocation sequence and prepared the envelopes, while the intervention administrator, who was not involved in baseline assessments, opened the envelopes and assigned participants to their respective groups. The intervention providers were not blinded due to the nature of the study [47]. Participants were blinded to the study hypothesis and were not informed about the other intervention groups. To ensure blinding, assessments were conducted by a single trained physical therapist who was not involved in the intervention. The assessor was unaware of the group allocation.

### Development of the Intervention (Experimental Groups)

The balance-based exergame training used in this study was developed through a structured, theory-driven process. A prior EEG-based study demonstrated that each difficulty level elicited distinct acute cognitive demands, thereby validating the capacity of the training framework to deliver graded cognitive load. These findings provided the rationale for stratifying participants by difficulty level in the present trial [38]. The intervention design was guided by the Design, Dynamics, and Experience framework, which helped structure gameplay elements across design mechanics, real-time interactions, and user experience [48]. Although end users were not directly involved in development, the Design, Dynamics, and Experience framework supported the integration of meaningful cognitive-motor challenges. Additionally, the intervention was grounded in core neuroplasticity principles, specificity, repetition, intensity, and salience, and aligned with motor learning frameworks to promote cognitive-motor engagement and adaptive outcomes [49]. The manipulation of cognitive task variables, such as dual-task components, attention demands, and response inhibition, was guided by the theoretical foundation of the Challenge Point Framework and motor learning theory, which advocate for optimizing task difficulty relative to individual capacity to enhance learning and neuroplastic adaptation [50].

Weight-shifting was selected as the core motor task due to its safety and feasibility for individuals with MCI, its role as a foundational balance skill essential for gait initiation, and its suitability for precise cognitive-motor manipulation without altering the primary motor task. This choice enabled controlled, progressive targeting of both balance and gait-related postural control in a clinically safe, evidence-informed manner. Details of the intervention parameters and training protocol are mentioned in Table 1, and intervention specifications are provided in Table 2.

**Table 1:** Intervention parameters and training protocol.

Parameter			Description
**Intervention parameters [51]**			
	Frequency (F)	Three supervised sessions on nonconsecutive weekdays (Monday, Wednesday, and Friday) per week for 8 weeks (total 24 sessions).	
	Intensity (I)	Fixed for each difficulty group and Wii Fit group throughout the intervention.	
	Time (T)	Forty minutes per session (5-minute warm-up, 30 minutes of exergame training, 5-minute cool-down).	
	Type (T)	Experimental groups: Android-based football-themed exergame on a wobble board. Control group: Wii Fit balance games (Soccer Heading, Penguin Slide, and Table Tilt).	
	Density (D)	Each session comprised five 6-minute rounds with a 5-minute rest after the third round (work-to-rest ratio 6:1).	
**Training protocol** **[39,52]**			
	Instructions to participants	All participants were thoroughly instructed at the start of the intervention regarding all these key aspects of the study protocol.	
	Setting and supervision	All sessions were center-based and fully supervised by experienced physical therapists >2 years of experience in neurorehabilitation, who underwent specific training in exergame delivery at Pakistan Railway Hospital.	
	Delivery of sessions	Sessions were conducted individually under supervision to ensure safety and correct execution.	
	Trial session	Each participant received a trial session regarding the demonstration of the setup and training procedure.	
	Standardized exergame	Exercises were standardized for each group (low, moderate, and high difficulty) and Wii Fit, not individually tailored.	
	Starting level decision rule	Participants were assigned to an exergaming group based on randomization rather than performance.	
	Safety strategy	A standing frame was positioned in front of each participant to ensure their safety during the intervention.	
	Adherence measurement	Attendance logs were maintained for all 24 sessions, and missed sessions were tracked and completed.	
	Motivation strategies	Therapists used verbal encouragement, progressive feedback, and goal setting (eg, achieving higher game scores) to motivate participants.	
	Home program component	The intervention did not include a home-based program; all training was conducted in the hospital.	
	Assessment time Points	Assessments were carried out at baseline, after 4 weeks, and 8 weeks.	
	Adverse events	Throughout the clinical trial, adverse events, including dizziness, nausea, vertigo, and musculoskeletal pain, were monitored and documented in a therapist’s log. Participants experiencing persistent symptoms were documented and subsequently excluded from the study.	

**Table 2:** Intervention details.

Groups	Experimental groups (low, moderate, and high difficulty)	Wii Fit group
Intervention protocol and training setup	Customized wobble board, measuring 40 cm in diameter and 10 cm in height from the apex to the top [38,53], with a designed space at the center to hold the mobile. The Android game was displayed on a 43” light-emitting diode (LED) screen. Experimental group game and setup are given in Figure ! [39].	Wii Fit Balance Board (WBB; Nintendo, Kyoto, Japan)
Games	An Android-based football exergame featuring 3 predefined difficulty levels. The game includes 8 goal spots with dynamic obstacles in front of each goal spot. Criteria for difficulty levels Low: Goal spot size:1 unit in length Ball speed: slow (the ball moves in 0.8 seconds) Moderate: Goal spot size: 0.8 units in length Ball speed: medium (the ball moves in 0.6 seconds) High: Goal spot size: 0.6 units in length Ball Speed: high (the ball moves in 0.4 seconds) [38,39].	Three balance training games: soccer heading, table tilt, and penguin slide
Intervention session plan (30 minutes)	Total rounds of football games: 5 rounds Games per round: 3 games Duration per game: 2 minutes Total duration per round: 6 minutes Rest interval: 5 minutes to prevent fatigue after the 3rd round (after 18 minutes) [39].	Soccer heading: 7 times (Time: 1 minute/game) Penguin slide: 7 games (Time: 1 minute/game) Table tilt: 8 games (Time: 2 minutes/game) Rest interval: 5 minutes to prevent fatigue after 2 sets of games (after 14 minutes) [39].
Game Task	The task is to score a goal on the highlighted target by shifting body weight on the wobbleboard in either direction to score more goals.	Soccer heading: Hit the ball quickly and accurately while avoiding obstacles. Penguin slide: The goal is to catch fish by tilting the iceberg while maintaining balance Table tilt: The goal is to move all the balls into the holes within a set time limit
Procedure	A novel android-based football-themed exergame, developed by Ghani et al [38], was used for the experimental groups. The game was specifically designed to provide graded cognitive-motor challenges through 3 predefined difficulty levels (low, moderate, and high). Participants stood on a custom-made wobble board and shifted their body weight to control the ball on the screen, aiming to score goals on highlighted targets while navigating dynamic obstacles. This setup created a dual-task environment, requiring balance control, postural adjustments, and visuomotor coordination. The training setup is illustrated in Figure 2.	The Wii Balance Board’s embedded electronic sensors detected subtle shifts in body weight and provided real-time visual feedback on the screen. The training consisted of 3 Wii Fit balance games—Soccer Heading, Table Tilt, and Penguin Slide. In Soccer Heading, participants shifted weight to head virtual reality–based soccer balls while dodging obstacles; in Penguin Slide, they rocked side-to-side to catch fish; and in Table Tilt, they leaned in multiple directions to guide balls into holes, requiring precise balance control.

**Figure 2:**
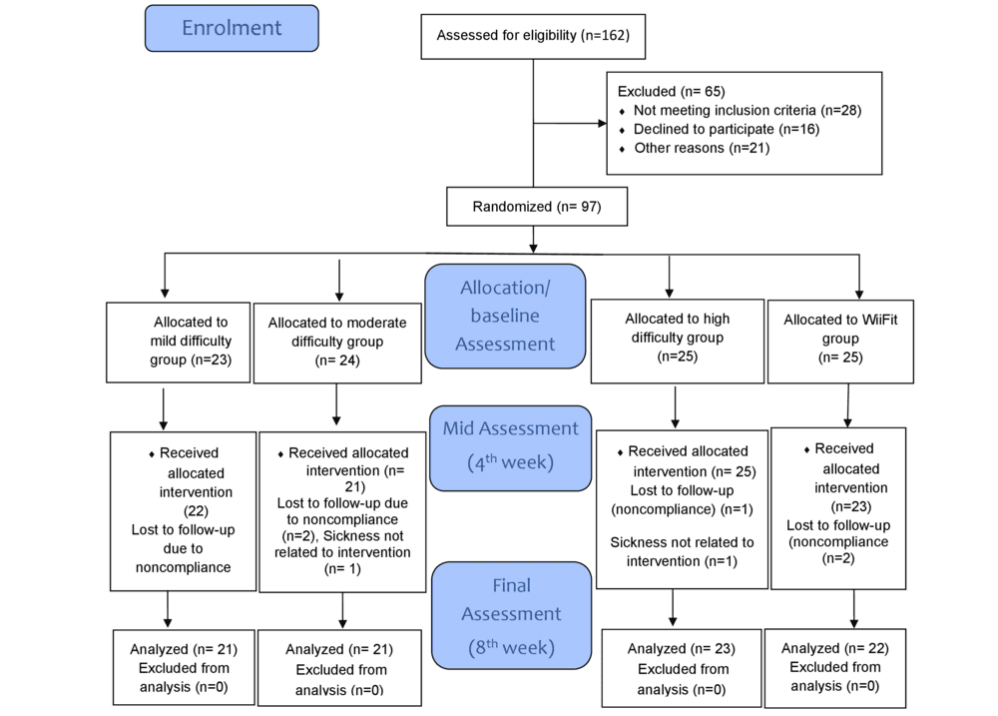
CONSORT (Consolidated Standards of Reporting Trials) flow diagram.

### Intervention of the Control Group

The control condition consisted of motor-cognitive exergaming using a recreational exergame (Wii Fit), which was selected as an active comparator rather than a passive control because it is validated for improving balance and incorporates cognitive-motor engagement [54]. This alignment reflects the current classification framework that distinguishes exergame-based motor-cognitive training (serious exergame), designed for rehabilitation purposes, from motor-cognitive exergaming (Wii Fit), which is primarily developed for entertainment. The use of Wii Fit allowed comparison of a purposefully designed, theory-driven serious exergame intervention with a widely available, recreational exergame, while ensuring participant engagement, reducing dropout risk, and providing an appropriate control group intervention.

### Measurement of Balance and Spatiotemporal Gait Parameters

Balance and gait outcomes were assessed by the evaluator using standardized clinical tools and a validated mobile app. The Mini-BESTest was administered as a screening measure of balance prior to enrolment [55]. Outcome measures included the timed up and go (TUG), cognitive time up and go (C-TUG), and the Gait & Balance (G&B) smartphone app. These instruments were selected for their well-established psychometric properties and clinical relevance in older adults [56-58]. Together, they provide a comprehensive evaluation of both static and dynamic balance, as well as spatiotemporal gait parameters, enabling a multidimensional assessment of mobility and fall risk.

### Clinical Balance Measures

#### TUG

It is a valid and reliable test for measuring functional mobility and fall risk in adults with MCI intraclass correlation coefficient 0.757-0.988) [56,59]. The TUG assessment required the participants to stand from a seated position, walk a distance of 3 meters, turn around, walk back, and return to a sitting position, quickly but safely [56]. The total duration to complete the activity was measured in seconds. The shorter duration represented better functional mobility [56].

#### C-TUG

It has high validity and intrarater reliability (intraclass correlation coefficient 0.91-0.94) in older adults with and without dementia [60,61]. The participants stood up from a seated position, walked 3 meters, and returned to the chair while serially counting backward by subtracting 3 loudly from 20 [57]. The time to complete the C-TUG was measured in seconds.

#### Gait & Balance Smartphone App (G&B App)

It is a valid and reliable mobile phone app that assesses balance and gait parameters by using inertial data from accelerometers and gyroscopes embedded into the smartphone [62]. For assessment purposes, the same iPhone 7 (Apple Inc) was used for all participants to ensure consistency of sensor-based measurements. The phone was strapped with the help of an elastic core stability belt within the customized phone case (Sports armband, Tech. Inc, Auckland, New Zealand) strapped in the lower lumbar spine, approximately L5/S1 level. The assessment protocol comprised 6 standardized tasks: 4 static balance tasks and 2 dynamic gait tasks. During the quiet stance, participants stood with feet hip-width apart and arms at their sides for 30 seconds while the app recorded postural sway in mediolateral (ML) and anteroposterior (AP) directions. Tasks included (1) standing on a hard surface with eyes open (FirmEO), (2) standing on a hard surface with eyes closed (FirmEC), (3) standing on a soft surface with eyes open (ComplaintEO), (4) standing on a soft surface with eyes closed (ComplaintEC). Dynamic gait tasks included (5) walking with looking forward (head forward [HF]), which involves walking while keeping the head and eyes facing straight ahead; (6) walking with head turns (HTs), which involves walking while actively turning the head side to side [62]. Each walking task was performed 4 times at a comfortable pace, with trials lasting 6 seconds. Auditory prompts (“ready, set, go,” “rest,” and “turn around”) standardized task execution. Testing was discontinued if participants lost balance, opened their eyes during eyes closed (EC) tasks, or required assistance [62]. The app extracted metrics such as postural sway, walking speed, step time, step length, and variability and asymmetry of step timing and length. Validation studies have demonstrated moderate-to-excellent convergent validity of the app with 3D kinematic systems for postural stability (r=0.98), step time (r=0.97), walking speed (r=0.79), and step length (r=0.73) [63]. Test-retest reliability for these parameters was also moderate to excellent. However, validity and reliability were poor for variability and asymmetry metrics, a limitation that was also evident in gold-standard systems and is attributed to low between-subject variance and brief trial durations [63].

### Important Changes to the Trial After Commencement

Few deviations from the initially registered clinical trial protocol were made to enhance methodological rigor and study quality.

1. We recruited 97 participants instead of the registered 90 to ensure adequate power in case of dropouts, although the original calculation required only 80 plus 20% for attrition.
2. Eligibility criteria were refined: the MoCA cut-off was set at <26 (MCI range 18-25) in line with current literature [64], and the Mini-BESTest replaced the Berg Balance Scale due to its broader assessment of anticipatory, reactive, sensory, and dynamic balance domains.
3. The intervention duration was increased from 30 to 40 minutes per session by adding warm-up and cool-down periods, ensuring alignment with best-practice exercise guidelines.

### Statistical Analysis

Data analysis was performed using SPSS Statistics for Windows (version 26; IBM Corp). The Kolmogorov-Smirnov test was conducted to assess normality. For TUG and C-TUG, the data were normally distributed. However, the data were nonnormal for all of the variables of the G&B mobile app except for HF walking speed, HT walking speed, and HT step time. Log transformation was applied to normalize the data with a nonnormal distribution. The Box-M test was used to assess the assumption of homogeneity, with results >0.05 for all variables. A mixed-model analysis of covariance (ANCOVA) with baseline values set as covariates was used to account for potential baseline differences [65,66]. The analysis determined the main effects of both “groups” and “time” as well as interaction effects between “groups” (low, moderate, high difficulty, and Wii Fit groups) and “time” (baseline, 4 weeks, and 8 weeks). The assumption of sphericity was assessed through Mauchley test of sphericity. Post-hoc analysis with Bonferroni correction was applied to adjust the significance level for multiple comparisons and reduce the risk of type I error [66]. ANCOVA-adjusted mean differences between groups were reported to account for baseline scores, and Bonferroni-corrected pairwise comparisons (Bonferroni-adjusted *t* tests) were then conducted to identify which groups differed significantly. A statistical significance level of 0.05 was established to test the null hypothesis, and the effect size was computed using η²ₚ (partial eta-squared) interpreted according to conventional benchmarks (η²ₚ; small=0.01, medium=0.06, large=0.14) [67].

## Results

### Overview

The calculated sample size was 80; however, to account for potential attrition, a total of 97 participants were initially recruited for the study. After 4 weeks (mid assessment), 92 participants completed the intervention (low difficulty: n=23; moderate difficulty: n=22; high difficulty: n=24; Wii Fit group: n=23), and by 8 weeks, 87 participants had completed the intervention (low difficulty: n=21; moderate difficulty: n=21; high difficulty: n=23; Wii Fit: n=22). This represented an overall attrition rate of 10%, well within the <20% threshold commonly cited for randomized controlled trials [68]. Completion adherence was 91.3% in the low-difficulty group, 87.5% in the moderate-difficulty group, 92.0% in the high-difficulty group, and 88.0% in the control group, indicating strong participant engagement across all arms. All participants randomized were included in the primary analysis according to their original assigned groups (intention-to-treat). Missing data due to dropout (10%) were addressed using the mixed model method, ensuring unbiased estimation and preservation of randomization integrity. The CONSORT flow diagram (Figure 2) illustrates participant recruitment, allocation, follow-up, and analysis, and demographic characteristics are provided in Table 3.

**Table 3:** Baseline demographic and clinical characteristics of participants across the groups.

Demographic variables and subcomponents		Exergaming group			
		Low difficulty	Moderate difficulty	High difficulty	Wii Fit
**Sex**					
	Male, n (%)	20 (20.61)	17 (17.52)	22 (22.68)	17 (17.52)
	Female, n (%)	3 (3.09)	7 (7.21)	3 (3.09)	8 (8.24)
Age (years), mean (SD)		72.52 (7.38)	64.83 (4.88)	69.72 (8.07)	72.32 (7.74)
BMI, mean (SD)		25.56 (5.71)	24.99 (2.95)	24.85 (3.77)	27.84 (5.36)
Mini BESTest^a^, mean (SD)		23.87 (2.94)	23.58 (3.53)	24.20 (2.87)	23.17 (3.14)

^a^Mini BESTest: Mini balance evaluation system.

### Clinical Balance Measures

#### Interaction Effect (Groups × Time)

**Figure 3:**
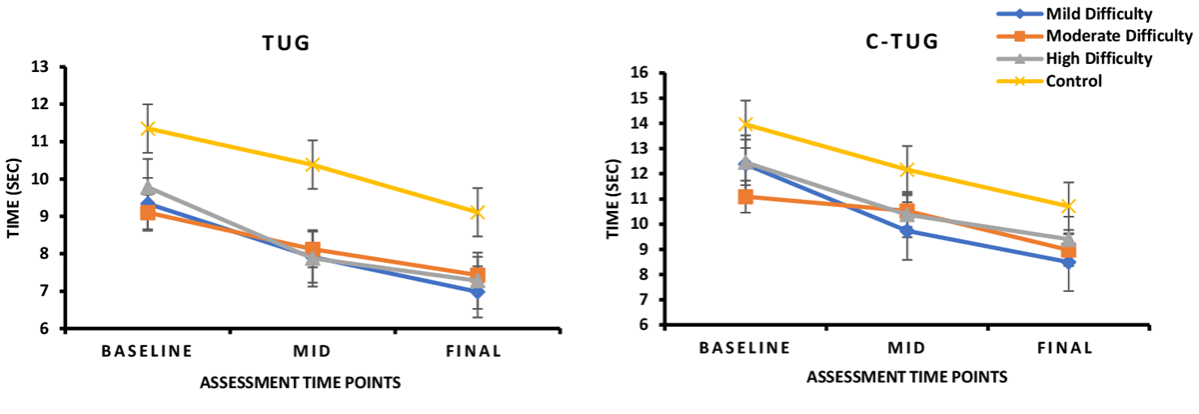
In this 8-week randomized controlled trial involving individuals with mild cognitive impairment, changes in TUG and C-TUG performance varied across intervention groups. Participants in the high-difficulty group demonstrated the greatest reduction in completion time for TUG, while participants in the low-difficulty group showed the greatest reduction in C-TUG time, indicating improved functional mobility in both groups compared with the Wii Fit control group (*P*<.05). C-TUG: cognitive time up and go; TUG: time up and go.

Significant group × time interactions were observed for TUG test (*F*_1,3_=19.34, *P*<.001, η²ₚ=0.217) and the C-TUG test (*F*_1,3_=21.24, *P*<.001, η²ₚ=0.233; Figure 3). These findings indicate that changes in performance over time varied significantly between intervention groups.

#### Post Hoc Pairwise Comparisons

Post hoc ANCOVA revealed that the high-difficulty group showed significantly greater improvements in TUG scores than the Wii Fit group (*P*<.05). For C-TUG, the low-difficulty group improved significantly more than the Wii Fit group (*P*<.05; Table S1 in Multimedia Appendix 3). These results suggest that higher difficulty training enhanced general mobility and dynamic balance, while lower difficulty training benefited mobility tasks involving greater cognitive engagement, such as dual-task walking.

### Static Stability

#### Interaction Effect (Groups × Time)

**Figure 4:**
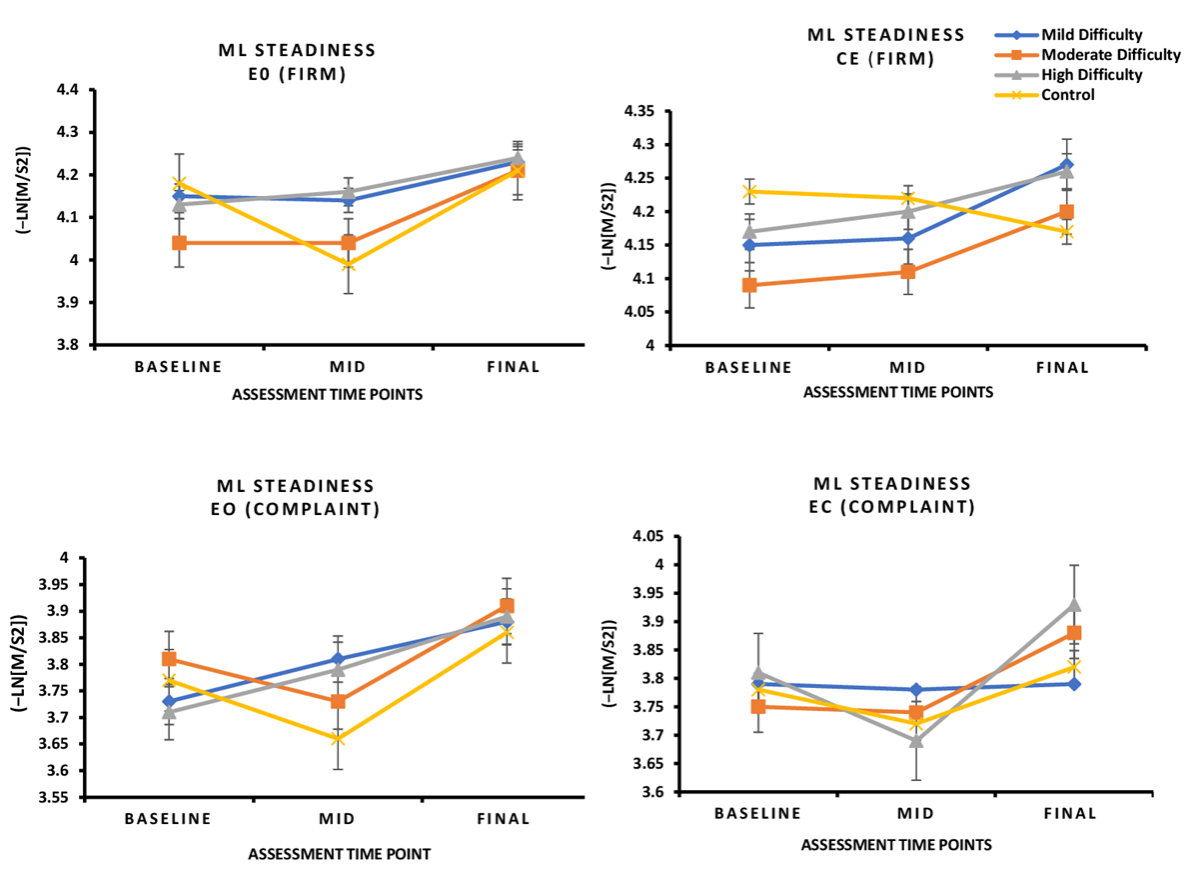
In this 8-week randomized controlled trial involving individuals with mild cognitive impairment, changes in ML steadiness under EO and EC conditions on firm and compliant surfaces did not vary across intervention groups. No significant between-group differences were observed in ML steadiness under either EO or EC conditions on firm or compliant surfaces (*P*>.05). EC: eyes closed; EO: eyes open; ML: mediolateral.

No significant group × time interaction effect was found for MLsteadiness during FirmEO, FirmEC, ComplaintEO, and ComplaintEC conditions (*P*>.05; Figure 4).

**Figure 5:**
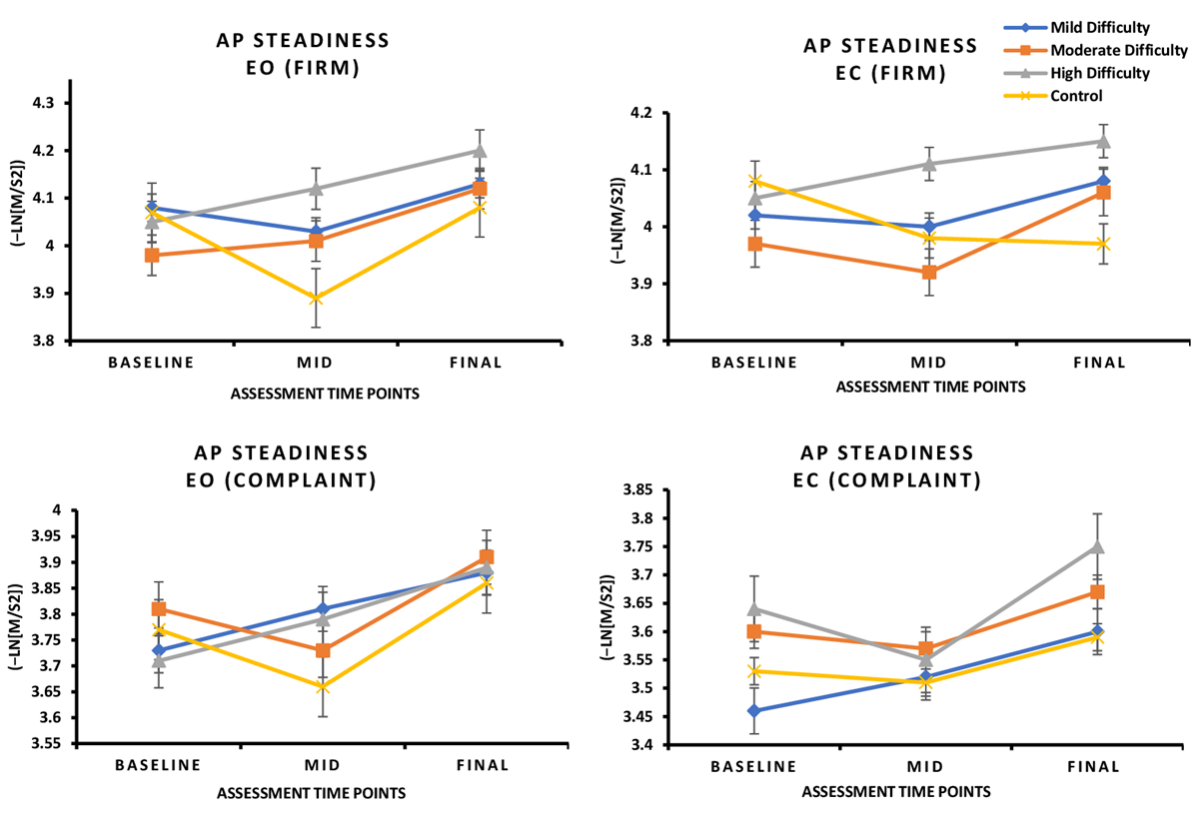
In this 8-week randomized controlled trial involving individuals with mild cognitive impairment, changes in AP steadiness under EO and EC conditions on firm and compliant surfaces varied across intervention groups. Participants in the low-, moderate-, and high-difficulty groups demonstrated a significant improvement in AP steadiness with EO on the compliant surface compared with the Wii Fit control group (*P*<.05). AP: anteroposterior; EC: eyes closed; EO: eyes open.

A significant group × time interaction was found for AP steadiness during the FirmEO condition (*F*_1,3_=4.270, *P*=.04, η²ₚ=0.066; Figure 5).

#### Post Hoc Pairwise Comparisons

Post hoc analysis showed that low‑, moderate‑, and high-difficulty groups all demonstrated significantly greater AP steadiness improvements compared with the Wii Fit group (*P*<.05; Table S2 in Multimedia Appendix 3). These findings indicate that balance control improved across all 3 exergame difficulty levels.

### Spatiotemporal Gait Parameters

#### Interaction Effect (Groups × Time)

**Figure 6:**
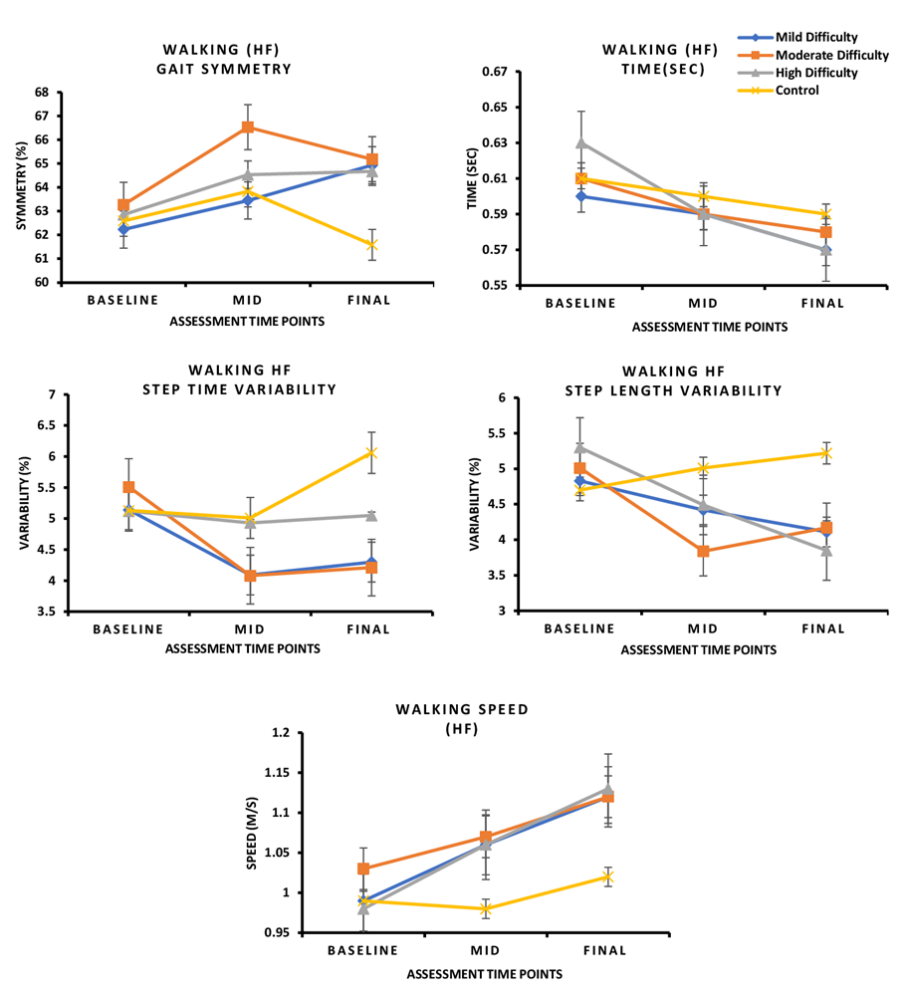
In this 8-week randomized controlled trial involving individuals with mild cognitive impairment, changes in walking parameters under HT conditions varied across intervention groups. Participants in the low- and high-difficulty groups showed a significant reduction in step time compared with the Wii Fit control group (*P*<.05). Moreover, the high-difficulty group demonstrated a significant reduction in step time variability and an improvement in walking speed relative to the Wii Fit group (*P*<.05). HF: head forward; HT: head turn.

For the walking with HF task, significant interaction effects were observed for step time (*F*_1,3_=6.117, *P*=.02, η²=0.093) and step time variability (*F*_1,3_=3.739, *P*=.05, η²ₚ=0.059; Figure 6).

**Figure 7:**
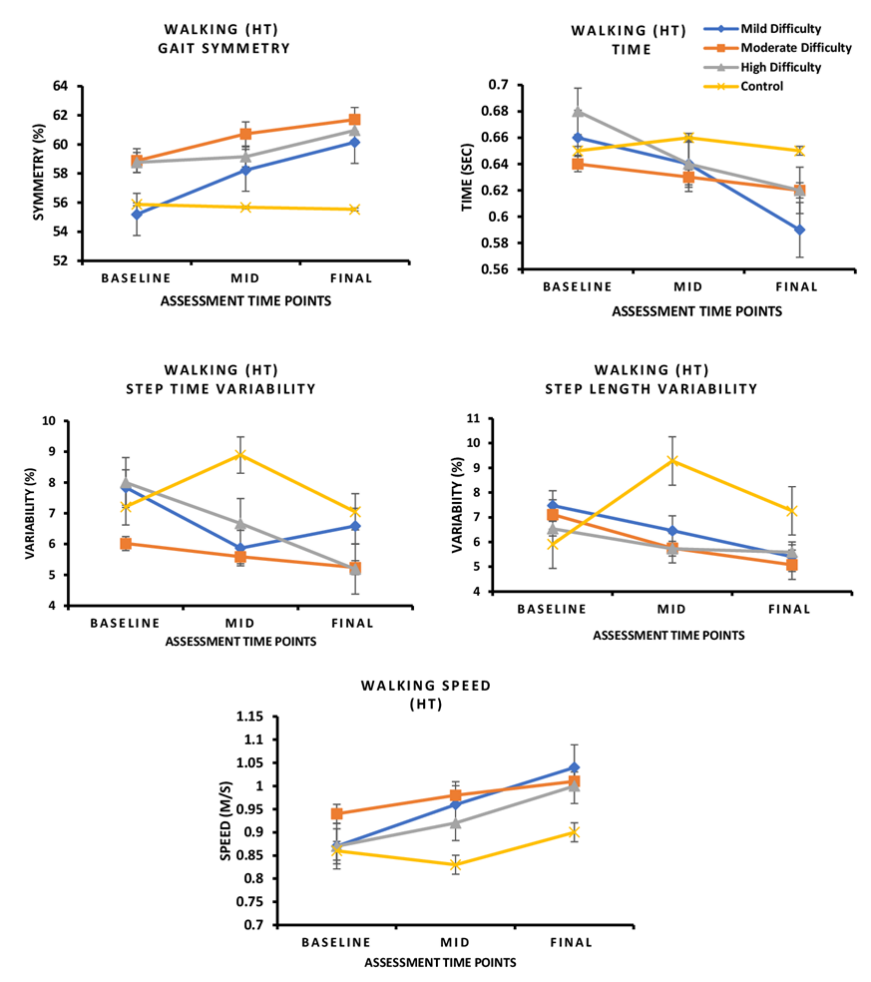
In this 8-week randomized controlled trial involving individuals with mild cognitive impairment, changes in walking parameters under HT conditions varied across intervention groups. Step time variability was significantly reduced across all difficulty groups compared with the Wii Fit control group (*P*<.05). In addition, the high-difficulty group demonstrated a significant reduction in step time (*P*<.05), while both the low- and high-difficulty groups showed significant reductions in step time variability and improvements in walking speed relative to the Wii Fit group (*P*<.05). HT: head turn.

For the walking with HT task, a significant interaction effect was found for step time variability (*F*_1,3_=3.739, *P*=.06, η²ₚ=0.059; Figure 7). These results suggest that different levels of exergame difficulty influenced step timing and gait stability over time.

#### Post Hoc Pairwise Comparisons

##### Walking With HF

The low- and high-difficulty groups showed significantly greater improvements in step time variability compared with the Wii Fit group (*P*<.05). The high‑difficulty group also demonstrated significant improvements in walking speed versus Wii Fit (*P*<.05; Table S3 in Multimedia Appendix 3).

##### Walking With HT

The high-difficulty group showed significant improvements in step time, step time variability, step length variability, and walking speed compared with the Wii Fit group (all *P*<.05). The moderate- and low-difficulty groups also improved step time variability, and the low-difficulty group demonstrated additional gains in walking speed compared with Wii Fit (*P*<.05; Table S3 in Multimedia Appendix 3).

## Discussion

### Principal Findings

To the best of our knowledge, the impact of balance-based exergame training involving graded cognitive-motor challenges on gait and balance functions in older adults with MCI has not been thoroughly assessed. The study showed significant improvement in dynamic balance (measured using TUG and C-TUG tests), as well as in AP steadiness with FirmEO condition. Improvement was also noted in gait parameters, including step time and step time variability under HF and HT conditions. The training was designed to enhance controlled weight-shifting in an exergaming environment, promoting both static and dynamic balance, alongside cognitive improvement. The effect of this intervention in enhancing various executive functions has already been reported, with more pronounced improvement in the high difficulty group [39].

### Clinical Balance Measures

This study identified significant improvement in TUG and C-TUG tests among participants in the low and high-difficulty groups, while the moderate-difficulty group showed comparatively less progress. This pattern can be understood through several theoretical frameworks in motor learning and rehabilitation science.

The differing outcomes across difficulty levels reflect that cognitive and motor learning have distinct optimal challenge thresholds. In the low-difficulty group, tasks were cognitively undemanding, allowing participants to allocate attentional resources to motor refinement, resulting in significant gains in balance and mobility tasks but little measurable cognitive improvement. This aligns with Fitts and Posner cognitive stage of motor learning [69], where reduced cognitive load supports the acquisition of correct movement patterns. In contrast, the moderate-difficulty group may have occupied a zone of challenge that is cognitively demanding enough to divide attention and increase mental load, but not sufficiently intense to trigger the deeper engagement and neuroplastic motor adaptations. By contrast, the high-difficulty group provided intense cognitive-motor demands and error-driven feedback, fulfilling key principles of experience-dependent neuroplasticity (specificity, salience, and challenge) [49]. This higher level of demand required participants to integrate motor planning, postural control, and rapid corrective adjustments, translating into improvements across both cognitive parameters and speed-related gait and fall-risk outcomes.

These findings align with previous research emphasizing that the cognitively challenging training enhanced balance performance [70,71]. Virtual reality–based cognitive training has been shown to improve cognitive function and performance in both TUG [25,70,72] and C-TUG tests [70]. Exergaming facilitates sensorimotor integration by enhancing the brain’s ability to process and integrate visual, vestibular, and proprioceptive inputs, leading to more efficient motor control [73]. The real-time visual feedback provided during exergaming aids in correct postural alignment and movement patterns, resulting in better coordination of muscle groups for specific motor tasks and ultimately contributing to improving TUG performance [72]. Moreover, exergaming on unstable surfaces introduces simultaneous multitasking, which enhances the brain’s capacity to manage motor and cognitive tasks concurrently, an essential aspect of executive functions. Regular engagement in these graded cognitive-motor challenges has been shown to improve dual-task performance, as observed in the C-TUG [74].

Interestingly, no significant differences in TUG and C-TUG improvements were observed across different difficulty groups overall. However, the group comparison indicated that the high-difficulty training group showed a better TUG performance than the Wii Fit group, suggesting that higher cognitive demands may be needed to effectively enhance mobility outcomes. In contrast, for C-TUG, a significant improvement was found between the low-difficulty and Wii Fit groups, suggesting that dual-task mobility improved most when participants trained under conditions of manageable cognitive load. According to cognitive load theory [75], tasks that do not overwhelm working memory allow attentional resources to be shared across motor and cognitive domains, supporting dual-task integration. By contrast, moderate and high difficulty tasks may have exceeded participants’ cognitive capacity, overloaded executive function, and limited gains in dual-task walking performance. An additional explanation may be related to attentional prioritization, whereby participants exposed to higher difficulty levels may have prioritized motor performance during training at the expense of cognitive performance. This would explain why improvements emerged primarily in single-task TUG but not in C-TUG performance, consistent with previous findings on prioritization effects in dual-task performance [76].

### Static Stability

A significant group × time interaction effect was observed for AP steadiness under the FirmEO condition, indicating a marked difference in AP stability among various difficulty levels of exergame balance training. AP movements, such as forward and backward shifts, are generally easier to control, as they engage larger muscle groups, including quadriceps and hamstrings [77]. In contrast, ML movements, which involve side-to-side shifts, require more intricate control from smaller stabilizing muscles, along with greater reliance on proprioceptive and vestibular systems [77]. Additionally, AP steadiness tends to improve more readily due to the anatomical alignment of the foot, which is optimized for efficient movement control in the forward-backward plane. This finding is consistent with previous research, which suggests VR training significantly enhances AP stability in adults with MCI [78]. However, a study with contradictory findings reported great improvement in ML stability compared with AP stability [79]. This difference may be explained by the higher initial instability in the ML direction observed in that study, along with the training conducted on a stable surface that emphasized side-to-side movements, which could account for the greater enhancement of ML stability relative to AP stability.

A meta-analysis revealed that individuals with MCI demonstrate greater postural sway while standing with eyes open (EO) compared with healthy controls. This may be attributed to impairments in visual processing and visuospatial integration; when these impairments are more serious, balance deficits become more pronounced, since vision is essential for achieving a stable posture [7]. The significant improvement in AP steadiness with EO condition observed in this study underscores the importance of visual feedback in postural control. With EO, participants continuously receive visual information about their body’s position, enabling precise and timely postural adjustments relative to the environment. This visual input also enhances the integration of sensory information from different systems, facilitating more rapid learning and contributing to the observed improvement in AP balance during exergaming [80]. On the other hand, in the EC condition, individuals must rely heavily on proprioceptive and vestibular inputs to maintain balance. In individuals with MCI, these systems may be less effective due to cognitive decline affecting sensory integration and processing, further challenging postural control. This impaired integration can make it more challenging to maintain AP and ML steadiness with eyes closed [80].

The post-hoc analysis demonstrated no significant difference between difficulty groups. Static steadiness tasks, which primarily assess balance in a fixed position, may not be sensitive enough to detect improvements in balance during less challenging tasks, as these tasks do not fully reflect the dynamic balance demands required in higher difficulty levels. This may explain the lack of significant differences in EC steadiness across difficulty levels. However, participants in the low, moderate, and high-difficulty groups showed significant improvement in AP steadiness under the EO condition compared with the Wii Fit group. These results suggest that higher difficulty exergaming effectively improves AP steadiness, likely due to the increased physical and cognitive demands of such exercises, which promote greater focus, coordination, and overall balance stability [31].

### Spatiotemporal Gait Parameters

A significant difference in improvement was observed among the groups after 8 weeks of intervention in step time and step time variability during waking under the HF conditions. Step time variability also differs across the groups under the HT condition, as indicated by mixed ANCOVA analysis. Gait is a multifaceted task requiring substantial attention, which involves physical capacity, balance, and cognitive control, including attention allocation, inhibition of distractions, and motor planning [81]. Integrating cognitive interventions, such as dual-tasking exergaming with cognitively challenging activities, may further enhance gait performance [33]. The literature supports these findings, showing significant improvement in gait speed with exergaming [31,33], and gait speed is widely recognized as one of the primary gait parameters positively influenced by exergaming [33]. Furthermore, step time variability serves as an important indicator of gait stability [33], suggesting that exergaming not only improves speed but also contributes to greater stability and control during walking.

The post-hoc analysis revealed differential improvements across groups in specific walking parameters under dual-task conditions. Notably, the high-difficulty condition yielded greater reductions in step time variability (in HF) and significantly faster walking speed (HF), as well as lower step time (HT), compared with the Wii Fit group. These results indicate that increased task challenge, combining cognitive load and physical demand, selectively enhanced motor control, consistency, and gait efficiency.

This aligns with findings from a recent randomized controlled trial and a meta-analysis, both of which reported that exergames incorporating greater postural and cognitive demands yield more pronounced improvements in gait and balance [32,82].

Furthermore, studies emphasize that exergames integrating cognitive-motor dual-tasking—especially in those with higher difficulty levels- produce gains in executive function and attentional control that mediate gait improvements [29]. Neurophysiological research has shown that increased frontal theta activity during higher-demand exergames reflects enhanced neural engagement of cognitive control networks essential to coordinate timing and balance [83]. These neurocognitive demands likely improved executive-motor integration, thereby supporting faster and more stable walking under HF/HT conditions [84].

Building on these findings, a differentiated pattern emerged across difficulty levels. The low- and high-difficulty exergaming groups achieved the most notable gains, though likely through different mechanisms. Low-difficulty tasks appeared to provide an accessible point for motor practice by reducing cognitive demands and minimizing dual-task interference, thereby enabling participants to consolidate core postural strategies and movement patterns [85,86]. By contrast, high-difficulty tasks placed greater demands on attention, motor planning, and adaptive control, which evidence suggests can stimulate neuromuscular activation, sensorimotor integration, and neuroplastic changes leading to improvements in gait variability and walking speed [32]. The moderate-difficulty condition, however, seemed to sit in a “grey zone,” neither simple enough to free attentional resources for basic motor learning nor challenging enough to drive significant motor adaptation [87]. Together, these findings highlight the importance of carefully selecting the exergame task difficulty, starting with manageable tasks to consolidate fundamentals, then advancing to higher-demand tasks to trigger more complex motor adaptations and maximize therapeutic benefit [32,87]. Although task difficulty in this study was not individualized, the observed response patterns across groups are consistent with the principles of the optimal challenge point framework, which suggests that different levels of task demand may elicit distinct motor and cognitive adaptations [88].

The study findings suggest that graded cognitive-motor challenging exergames may be a valuable approach to targeting balance and gait deficits, emphasizing the potential for these training programs to optimize functional outcomes. The results also suggest that incorporating balance-focused exergame training with graded difficulty levels can enhance balance and gait performance in older adults with MCI, and potentially extend to other populations experiencing cognitive-motor impairments, such as individuals with other neurological conditions. These potential benefits highlight the clinical relevance and impact of this intervention [89]. Clinicians may consider incorporating such interventions alongside conventional therapy to enhance dual-task performance and potentially reduce fall risk in this vulnerable and fall-prone population [90]. Because exergame difficulty can be adjusted, therapists can tailor challenges to individual capabilities and progressively modify tasks to maintain optimal engagement, making this approach suitable for use in outpatient clinics.

### Limitations

The study had some limitations. First, the 8-week intervention duration may have restricted the overall impact of the training, and extending the intervention duration could potentially reveal more significant differences between various difficulty levels. Although adherence was closely monitored to ensure full exposure to the planned dose, longer interventions in future studies may reveal stronger or more sustained effects. Second, since the majority of the participants were male, the findings may not be fully generalizable to both sexes. Improvement in balance and gait differs between males and females [91], and this difference may persist when exposed to varying levels of exergaming difficulty. Recruiting a more sex-balanced sample is essential to enhance the generalizability of findings and to explore potential sex-based differences. Third, the C-TUG test used the same starting number and subtraction pattern throughout the study. This design may have introduced familiarity effects, potentially reducing the test’s sensitivity to detect subtle changes in dual-task performance and cognitive-motor interference across groups. Moreover, cognitive task performance (ie, number of correct responses) was not recorded during the C-TUG, preventing evaluation of possible dual-task trade-offs where mobility improvements could occur at the expense of cognitive accuracy or vice versa. Future studies should vary the secondary task and record accuracy to capture dual-task interference better.

Fourth, the intervention used a fixed design, although tasks were structured at low, moderate, and high levels; no progression mechanism was applied across the 24 sessions. It is therefore possible that motor or cognitive learning reduced the internal load over time, limiting the challenge necessary to trigger optimal neuroplastic adaptations. Future research should integrate progressive overload principles to maintain optimal challenge. Fifth, no objective or subjective measures of task demand or internal loading metrics (eg, perceived exertion or cognitive load) were calculated, limiting the ability to confirm whether the intended challenge levels were achieved for each individual. Future studies should incorporate these assessments to strengthen intervention validation. Six, as the trial was powered on cognition rather than motor outcomes, it is possible that some gait or balance effects were underpowered; future trials may consider sample size estimation directly based on these parameters. Seven, while the G&B app is valid for core gait measures, its reliability for step time variability and asymmetry is limited. Findings related to these variables should therefore be viewed in light of their known measurement limitations.

### Implications for Research

Future studies should test difficulty-graded exergames in broader clinical populations (eg, Parkinson disease and poststroke) and examine their long-term effects. Incorporating objective and subjective measures of task demand (eg, perceived exertion and cognitive load) will enable difficulty to be tailored to individual capacity and adjusted progressively, providing clearer insight into optimal dosing. Research should also evaluate how these interventions can be embedded into fall-prevention and healthy aging programs to maximize their impact on functional decline, independence, and accessibility across clinical, community, and home settings. The future work should also explore the integration of step-based training components alongside weight-shifting tasks to enhance gait adaptability, balance-gait synergy, and ecological validity, particularly in populations at risk of mobility decline.

### Conclusions

In conclusion, the 8-week exergame balance training effectively improved balance and spatiotemporal gait measures, including TUG, C-TUG, and AP stability on a firm surface, as well as step time and step time variability during walking under HF and HT conditions. However, no significant differences in improvement were observed across different difficulty groups for clinical balance measures, stability, and gait parameters. Both high-difficulty and low-difficulty groups showed improvement in stability, step time, and step length variability. Moreover, step time and walking speed with HF and HT significantly improved in the high-difficulty group. These findings suggest that exergame-based interventions with varying difficulty levels may enhance balance and gait parameters in older adults with MCI.

## Appendix

Multimedia Appendix 1 CONSORT 2010 checklist.

Multimedia Appendix 2 CONSORT-eHEALTH checklist (V 1.6.1).

Multimedia Appendix 3 Between-group differences in difficulty levels for clinical balance measurement, during quiet stance, and during walking.

## Abbreviations

ANCOVA: analysis of covariance
AP: anteroposterior
CONSORT: Consolidated Standards of Reporting Trials
CONSORT-EHEALTH: Consolidated Standards of Reporting Trials of Electronic and Mobile Health Applications and Online Telehealth
C-TUG: cognitive time up and go
EC: eyes closed
EO: eyes open
G&B: Gait & Balance
HF: head forward
HT: head turn
MCI: mild cognitive impairment
Mini-BESTest: Mini-Balance Evaluation Systems Test
ML: mediolateral
MoCA: Montreal Cognitive Assessment
TUG: time up and go

## Data Availability

The datasets generated or analyzed during this study are available from the corresponding author on reasonable request.
