# Supplementary material for: Effects of Balance-Based Exergame Training With Variable Difficulty on Balance and Spatiotemporal Gait Outcomes in Adults With Mild Cognitive Impairment: Randomized Controlled Trial
Source: JMIR Serious Games. 2025 Nov 18;13:e74092. doi: 10.2196/74092 (PMC12673310; doi:10.2196/74092)
Supplement: Multimedia Appendix 3 [file games_v13i1e74092_app3.docx]

***Table S1. Group difficulty level differences for clinical balance measure***

| **Qual** | **Parameters** | **GROUPS** | | | | | |
| --- | --- | --- | --- | --- | --- | --- | --- |
|  |  | **Low – Moderate**  **Difficulty** | **Moderate – High**  **Difficulty** | **Low– High**  **Difficulty** | **Low**  **Difficulty –Wii Fit** | **Moderate**  **Difficulty –Wii Fit** | **High**  **Difficulty–Wii Fit** |
| **TUG** | **Mean Difference± SE [95% CI]** | -0.34±0.32  [-0.98-0.30] | 0.47±0.30  [-0.14-1.07] | 0.13±0.31  [-0.49-0.75] | -0.58±0.34  [-1.26-0.09] | -0.24±0.33  [-0.91-0.42] | -0.71±0.32  [-1.34- -0.08] |
|  | **P-Value, Effect Size** | 0.293, 0.372 | 0.127, **0.391** | 0.686, **0.044*** | 0.089, 0.433 | 0.465, 0.122 | 0.028*,0.563 |
| **C-TUG** | **Mean Difference± SE [95% CI]** | -0.82±0.64  [-2.09- -0.46] | 0.19±0.61  [-1.03-1.41] | -0.63±0.59  [-1.82-0.56] | -1.61±0.63  [-2.86- -0.36] | -0.79±0.65  [-2.07-0.49] | -0.97±0.59  [-2.16-0.22] |
|  | **P-Value, Effect Size** | 0.204, 0.301 | 0.762, 0.025 | 0.293, 0.230 | 0.013*, 0.631 | 0.226, 0.279 | 0.108, 0.414 |

Timed Up and Go (TUG) and Cognitive Timed Up and Go (C-TUG) performance across intervention groups (Low, Moderate, High difficulty, and Wii Fit control). (Low, Moderate, High difficulty, and Wii Fit). Effect Size= η2 (small = 0.01, medium = 0.06, large = 0.14) *= p ≤ 0.05

***Table S2. Differences between groups' difficulty levels during quiet stance***

| **Outcomes** | **Task** | **Qual** | **Parameters** | **GROUP** | | | | | |
| --- | --- | --- | --- | --- | --- | --- | --- | --- | --- |
|  |  |  |  | **Low– Moderate**  **Difficulty** | **Moderate – High**  **Difficulty** | **Low– High**  **Difficulty** | **Low**  **Difficulty–**  **Wii Fit** | **Moderate Difficulty–**  **Wii Fit** | **High**  **Difficulty–**  **Wii Fit** |
| **ML steadiness (*−*ln[m/s2])** | **Firm** | **EO** | **Mean Difference± SE [95% CI]** | -0.00±0.02  [-0.06-0.04] | 0.00±0.03  [-0.05-0.06] | -.001±0.03  [-0.05-0.05] | 0.04±0.03  [-0.01-0.09] | 0.05±0.03  [-0.01-0.10] | 0.04±0.03  [-0.01-0.09] |
|  |  |  | **P-Value** | 0.750, **0.00** | 0.799, **0.00** | 0.954, 0.003 | 0.146, 0.042 | 0.109, 0.063 | 0.150, 0.042 |
|  |  | **EC** | **Mean Difference± SE [95% CI]** | -0.01±0.02  [-0.05-0.03] | 0.03±0.02  [-0.02-0.07] | -0.01±0.02  [-0.04-0.04] | 0.02±0.02  [-0.01-0.06] | 0.03±0.02  [-0.01-0.07] | 0.01± 0.02  [-0.03-0.05] |
|  |  |  | **P-Value, Effect Size** | 0.549, 0.006 | 0.288, 0.051 | 0.978, 0.006 | 0.239, **0.024** | 0.113,0.052 | 0.605, **0.006** |
|  | **Complaint** | **EO** | **Mean Difference± SE [95% CI]** | 0.01±0.02  [-0.02-0.05] | 0.00±0.02  [-0.03-0.04] | 0.02±0.02  [-0.02-0.05] | 0.00±0.02  [-0.03-0.04] | -0.01±0.02  [-0.05-0.03] | -0.02±0.02  [-0.05-0.02] |
|  |  |  | **P-Value, Effect Size** | 0.466, 0.006 | 0.745, **0.000** | 0.264, 0.023 | 0.842, **0.000** | 0.626, **0.006** | 0.403, **0.022** |
|  |  | **EC** | **Mean Difference± SE [95% CI]** | -0.03±0.02  [-0.07-0.01] | 0.01±.020  [-0.03-0.05] | -0.02±0.02  [-0.05-0.02] | -0.01±0.02  [-0.05-0.03] | 0.02±0.02  [-0.03-0.06] | 0.00±0.02  [-0.04-0.04] |
|  |  |  | **P-Value, Effect Size** | 0.162, 0.053 | 0.568, 0.006 | 0.398, 0.023 | 0.552, 0.006 | 0.458, **0.024** | 0.836, 0.000 |
| **AP steadiness (*−*ln[m/s2])** | **Firm** | **EO** | **Mean Difference± SE [95% CI]** | -0.03±.02  [-0.070-0.012] | 0.01±0.02  [-0.04-0.05] | -0.02±0.02  [-0.06-0.02] | 0.03± 0.02  [-0.02-0.06] | 0.05±0.02  [0.01-0.09] | 0.04±0.02  [0.00-0.09] |
|  |  |  | **P-Value, Effect Size** | 0.162,0.053 | 0.810, 0.006 | 0.218, 0.023 | 0.048*,0.052 | 0.029*, 0.132 | 0.039*,0.085 |
|  |  | **EC** | **Mean Difference± SE [95% CI]** | -0.04±0.03  [-0.10-0.02] | 0.02±0.03  [-0.05-0.08] | -0.03±0.03  [-0.08-0.03] | 0.01±0.03  [-0.05-0.07] | 0.05±0.03  [-0.01-0.12] | 0.03±0.03  [-0.03-0.09] |
|  |  |  | **P-Value, Effect Size** | 0.148, 0.043 | 0.574, 0.011 | 0.362, 0.023 | 0.795, 0.003 | 0.119, 0.0635 | 0.277, 0.023 |
|  | **Complaint** | **EO** | **Mean Difference± SE [95% CI]** | 0.00±0.02  [-0.03-0.03] | -0.00±0.02  [-0.03-0.03] | -0.00±0.02  [-0.03-0.03] | 0.01±0.02  [-0.03-0.04] | 0.00±0.02  [-0.03-0.04] | 0.01±0.02  [-0.03-0.04] |
|  |  |  | **P-Value, Effect Size** | 0.959,0.000 | 0.923, 0.000 | 0.959, 0.000 | 0.769, **0.006** | 0.824, 0.000 | 0.741, 0.006 |
|  |  | **EC** | **Mean Difference± SE [95% CI]** | -0.02±0.02  [-0.06-0.025] | 0.01±0.02  [-0.04-0.06] | -0.01±0.02  [-0.052-0.03] | 0.01±0.02  [-0.03-0.06] | 0.03±.02  [-0.02-0.08] | 0.02±0.02  [-0.03-0.07] |
|  |  |  | **P-Value, Effect Size** | 0.374**, 0.024** | 0.636, 0.006 | 0.674**, 0.006** | 0.590**. 0.006** | 0.189, 0.052 | 0.365, 0.023 |

Mediolateral (ML) and Anteroposterior (AP) steadiness (−ln[m/s²]) under Eyes Open (EO) and Eyes Closed (EC) conditions on firm and compliant surfaces across intervention groups (Low, Moderate, High difficulty, and Wii Fit). Effect Size= η2 (small = 0.01, medium = 0.06, large = 0.14) *= p ≤ 0.05

***Table S3. Differences between groups' difficulty levels during walking***

| **Task** | **Qual** | **Parameters** | **GROUPS** | | | | | |
| --- | --- | --- | --- | --- | --- | --- | --- | --- |
|  |  |  | **Low– Moderate Difficulty** | **Moderate-High Difficulty** | **Low –High Difficulty** | **Low Difficulty–Wii Fit** | **Moderate Difficulty–Wii Fit** | **High Difficulty–Wii Fit** |
| **HF** | **Gait symmetry (%)** | **Mean Difference± SE [95% CI]** | -0.00±0.02  [-0.04-0.03] | 0.03±0.02  [-0.01-0.06] | 0.02±0.02  [-0.01-0.05] | 0.01±0.02  [-0.02-0.05] | 0.02±0.02  [-0.02-0.05] | -0.01±0.02  [-0.04-0.02] |
|  |  | **P-Value, Effect Size** | 0.853, 0.000 | 0.119, 0.051 | 0.134, 0.023 | 0.408**, 0.006** | 0.351, 0.023 | 0.555, **0.0061** |
|  | **Step time (s)** | **Mean Difference± SE [95% CI]** | -0.01±0.02  [-0.06-0.04] | 0.03±0.02  [-0.02-0.08] | -0.02±0.02  [-0.03-0.06] | -0.00±0.02  [-0.06-0.03] | -0.01±0.03  [-0.06-0.04] | -0.00±0.02  [-0.06-0.03] |
|  |  | **P-Value, Effect Size** | 0.695, **0.006** | 0.245, 0.0508 | 0.383, 0.023 | 0.400, 0.000 | 0.679, 0.003 | 0.400, 0.000 |
|  | **Step time variability (%)** | **Mean Difference± SE [95% CI]** | -0.03±0.09  [-0.21-0.15] | -0.24±0.09  [-0.42- -0.05] | -0.27±0.08  [-0.44- -0.09] | -0.20±0.09  [-0.38--0.02] | -0.17±0.09  [-0.37--0.02] | 0.06±0.09  [-0.12-0.25] |
|  |  | **P-Value, Effect Size** | 0.740, 0.003 | 0.065, 0.145 | 0.760, 0.217 | 0.027*, 0.102 | 0.079, 0.080 | 0.020*, 0.107 |
|  | **Step length variability (%)** | **Mean Difference± SE [95% CI]** | -0.01±0.09  [-0.19-0.16] | -0.08±0.09  [-0.26-0.11] | -0.09±0.09  [-0.26-0.08] | -0.14±0.09  [-0.32-0.04] | -0.13±0.09  [-0.32-0.07] | -0.05±0.09  [-0.23-0.14] |
|  |  | **P-Value, Effect Size** | 0.876, **0.003** | 0.394, 0.019 | 0.280, 0.023 | 0.120, **0.056** | 0.194, 0.048 | 0.607, 0.007 |
|  | **Walking speed (m/s)** | **Mean Difference± SE [95% CI]** | 0.02±0.04  [-0.07-0.10] | -0.05±0.04  [-0.14-0.04] | -0.03±0.04  [-0.11-0.05] | 0.05±0.04  [-0.03-0.14] | 0.04±0.05  [-0.06-0.13] | 0.08±0.04  [-0.00-0.17] |
|  |  | **P-Value, Effect Size** | 0.650, 0.006 | 0.279, **0.037** | 0.477, 0.013 | 0.204, 0.036 | 0.450, **0.015** | 0.050*, 0.085 |
| **HT** | **Gait symmetry (%)** | **Mean Difference± SE [95% CI]** | 0.00±0.05  [-0.09-0.09] | -0.00±0.05  [-0.09-0.09] | 0.00±0.04  [-0.01-0.09] | 0.09±0.05  [-0.01-0.18] | 0.09±0.05  [-0.02-0.19] | 0.08±0.05  [-0.01-0.18] |
|  |  | **P-Value, Effect Size** | 0.971, 0.000 | 0.987, 0.000 | 0.983, 0.000 | 0.062, 0.073 | 0.095, 0.073 | 0.076, 0.057 |
|  | **Step time (s)** | **Mean Difference± SE [95% CI]** | -0.03±0.02  [-0.06-0.01] | 0.03±0.02  [-0.00-0.07] | 0.01±0.02  [-0.03-0.04] | -0.03±0.02  [-0.07-0.00] | -0.00±0.02  [-0.04-0.03] | -0.04±0.02  [-0.07-0.00] |
|  |  | **P-Value, Effect Size** | 0.125, 0.051 | 0.076, 0.051 | 0.735**, 0.006** | 0.072, 0.051 | 0.813, 0.000 | 0.042***, 0.087** |
|  | **Step time variability (%)** | **Mean Difference± SE [95% CI]** | -0.09±0.09  [-0.26-0.09] | -0.02±0.09  [-0.19-0.16] | -0.10±0.08  [-0.27-0.06] | -0.25±0.09  [-0.52- -0.18] | -0.26±0.09  [-0.45- -0.08] | -0.35±0.09  [-0.42- -0.06] |
|  |  | **P-Value, Effect Size** | 0.339, 0.024 | 0.828, 0.001 | 0.214, 0.037 | 0.008*, 0.158 | 0.007*, 0.169 | 0.000*, 0.260 |
|  | **Step length variability (%)** | **Mean Difference± SE [95% CI]** | -0.11±0.12  [-0.36- -0.14] | 0.07±0.13  [-0.18-0.32] | -0.04±0.12  [-0.028-0.19] | -0.31±0.12  [-0.55- -0.07] | -0.19±0.13  [-0.47-0.07] | -0.27±0.13  [-0.53- -0.01] |
|  |  | **P-Value, Effect Size** | 0.374, **0.021** | 0.575, 0.007 | 0.740, 0.003 | 0.014*, 0.137 | 0.139, 0.048 | 0.039*, 0.095 |
|  | **Walking speed (m/s)** | **Mean Difference± SE [95% CI]** | 0.06±0.04  [-0.03-0.14] | -0.04±0.04  [-0.12-0.04] | 0.02±0.04  [-0.06-0.09] | 0.09±0.04  [0.03±0.19] | 0.05±0.04  [-0.03-0.14] | 0.11±0.04  0.01-0.18] |
|  |  | **P-Value, Effect Size** | 0.168, 0.053 | 0.340, **0.015** | 0.669, 0.006 | 0.028*,0.105 | 0.225, 0.036 | 0.008*, 0.153 |

Spatiotemporal gait parameters (gait symmetry, step time, step time variability, step length variability, and walking speed) during habitual (HF) and high-task (HT) conditions across intervention groups (Low, Moderate, High difficulty, and Wii Fit). Effect Size= η2 (small = 0.01, medium = 0.06, large = 0.14) *= p ≤ 0.05

Spatiotemporal gait parameters (gait symmetry, step time, step time variability, step length variability, and walking speed) during habitual (HF) and high-task (HT) conditions across intervention groups (Low, Moderate, High difficulty, and Wii Fit). No significant group differences were observed for gait symmetry, step time, or step length variability under either HF or HT conditions (p > 0.05). Significant improvements were observed in step time variability (HF: Low vs Wii Fit, p = 0.027; High vs Wii Fit, p = 0.020) and in walking speed (HF: High vs Wii Fit, p = 0.050). Under HT conditions, significant between-group differences were found for step time (High vs Wii Fit, p = 0.042), step time variability (Low vs Wii Fit, p = 0.008; Moderate vs Wii Fit, p = 0.007; High vs Wii Fit, p < 0.001), step length variability (Low vs Wii Fit, p = 0.014; High vs Wii Fit, p = 0.039), and walking speed (Low vs Wii Fit, p = 0.028; High vs Wii Fit, p = 0.008), indicating superior gait stability and efficiency in intervention groups compared to control.
